# Integrative omics identification, evolutionary and structural analysis of low affinity nitrate transporters in diatoms, diNPFs

**DOI:** 10.1098/rsob.200395

**Published:** 2021-04-07

**Authors:** Anna Santin, Luigi Caputi, Antonella Longo, Maurizio Chiurazzi, Maurizio Ribera d'Alcalà, Monia Teresa Russo, Maria Immacolata Ferrante, Alessandra Rogato

**Affiliations:** ^1^ Stazione Zoologica Anton Dohrn, Villa Comunale, 80121 Naples, Italy; ^2^ BioDiscovery Institute, Denton, TX, USA; ^3^ Department of Biological Sciences, University of North Texas, Denton, TX, USA; ^4^ Institute of Biosciences and BioResources, CNR, Via P. Castellino 111, 80131 Naples, Italy

**Keywords:** diatoms, nitrogen, transporters, metagenomics, gene phylogeny

## Abstract

Diatoms are one of the major and most diverse groups of phytoplankton, with chimeric genomes harbouring a combination of genes of bacterial, animal and plant origin. They have developed sophisticated mechanisms to face environmental variations. In marine environments, nutrients concentration shows significant temporal and spatial variability, influencing phytoplankton growth. Among nutrients, nitrogen, present at micromolar levels, is often a limiting resource. Here, we report a comprehensive characterization of the Nitrate Transporter 1/Peptide Transporter Family (NPF) in diatoms, diNPFs. NPFs are well characterized in many organisms where they recognize a broad range of substrates, ranging from short-chained di- and tri-peptides in bacteria, fungi and mammals to a wide variety of molecules including nitrate in higher plants. Scarce information is available for diNPFs. We integrated-omics, phylogenetic, structural and expression analyses, to infer information on their role in diatoms. *diNPF* genes diverged to produce two distinct clades with strong sequence and structural homology with either bacterial or plant NPFs, with different predicted sub-cellular localization, suggesting that the divergence resulted in functional diversification. Moreover, transcription analysis of *diNPF* genes under different laboratory and environmental growth conditions suggests that diNPF diversification led to genetic adaptations that might contribute to diatoms ability to flourish in diverse environmental conditions.

## Graphical abstract

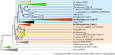

## 1. Introduction

Together with light availability, nutrient supply governs the life and distribution of photosynthetic organisms. Among the main macronutrients, there are nitrogen (N) compounds, available mostly as the inorganic ions ammonium (${\mathrm{NH}}_{4}^{+}$) and nitrate (${\mathrm{NO}}_{3}^{-}$). ${\mathrm{NO}}_{3}^{-}$, which serves both as an essential nutrient and a signal molecule in plants [1,2], represents the major bioavailable N source, but its availability fluctuates in both time and space. Land plants have evolved an extremely efficient system represented by the high- and the low- affinity Nitrate Transporter families (NRT2s and NRT1s, respectively), for sensing the external concentrations as well as the endogenous nutritional demand, uptake, distribution, storage and re-mobilization of nitrate [3,4]. NRT1s have recently been renamed as NPFs (NRT1/PTR) in plants [5].

Plant NPFs are phylogenetically related to a family of peptide transporters (PTRs) or proton-coupled oligopeptide transporters (POTs) that are evolutionarily conserved in archaea, bacteria, fungi and animals [6]. A remarkable feature of the POT/PTR/NPF family is the diversity and extent of natural substrates they recognize. These can be short-chained di/tri-peptides in bacteria, fungi and mammals, or a wide variety of molecules in higher plants, such as ${\mathrm{NO}}_{3}{}^{-}$ [5], nitrite (${\mathrm{NO}}_{2}{}^{-}$), di/tri-peptides, amino acids, dicarboxylates, glucosinolates [7,8] and phytohormones, as auxin (IAA) and abscisic acid (ABA) [9,10].

Although NPFs and POTs have evolved to transport a variety of substrates, structural studies have shown that overall they share the same structure, the canonical Major Facilitator Superfamily (MFS) fold. This fold consists of 12 transmembrane helices (TMH) organized into two six-helical bundles, with the N-terminal domain that includes TMH1–6 and the C-terminal domain with TMH7–12 [6,11–14]. Both N- and C-termini are located at the cytosolic membrane side. The substrate binding site is located in a clearly defined cavity that extends from the middle of the proteins towards the intracellular space.

POTs and most NPFs are substrate-proton symporters and are thought to use an alternating-access mechanism [15,16]. In this mechanism, transport is initiated when the protein is in the outward-open conformation by protons binding to chargeable amino acids belonging to the conserved ExxER motif on TMH1. This conformation is stabilized by a salt bridge formed by oppositely charged residues on TMH4 and TMH10, which also serves as an intracellular gate. After entry of the substrate, a series of conformational changes results in the breaking of the salt bridge, in the opening of the transporter's cavity towards the cytoplasm, and finally the release of the protons and the substrate.

This kind of transporters is present in a low number of copies in bacteria (1 to 4), in yeast (1), in animals (3 in *Drosophila melanogaster* and *Caenorhabditis elegans*, 4 in human), in green algae (1 in *Chlamydomonas reinhardtii* and *Micromonas pusilla,* 2 in *Coccomyxa subellipsoidea* and *Chlorella variabilis*), with some algae that do not present these transporters at all, like *Chlorella paradoxa* [17]. By contrast, in the plant genomes, there is a high number of NPFs, from the lowest of 20 members in *Physcomitrella patens* up to 139 in *Malus domestica* [5].

In marine species, the presence of low-affinity ${\mathrm{NO}}_{3}^{-}$ transporters is actually puzzling, since in the oceans ${\mathrm{NO}}_{3}^{-}$ concentrations are always lower than the approximate concentration (1 mM) at which NPFs become efficient, suggesting that either they are used for internal transport where local ${\mathrm{NO}}_{3}^{-}$ levels are higher, or that they are needed to transport different substrates than ${\mathrm{NO}}_{3}^{-}$ [18,19]. However, at least for *Arabidopsis thaliana* NPF6.3 and *Oriza sativa* OsNPF6.5, a dual affinity for ${\mathrm{NO}}_{3}^{-}$ has been demonstrated and in several cases the capacity of low-affinity NPFs to transport ${\mathrm{NO}}_{3}^{-}$ even in a range of concentrations extending from 10 µM to 500 µM has been reported [4,20–23].

Diatoms represent one of the dominant components of phytoplankton, contributing up to 40% of marine primary production [24]. Characterized by complex evolutionary history and chimeric genomes with plant-like, animal-like and bacterial traits [25–27], diatoms developed a unique metabolism, allowing them to adapt to changes in environmental stress conditions [28]. Diatoms are indeed considered as opportunistic (r-strategist) able to outcompete other phytoplankton in waters at high Si:P and Si:N ratios [29,30].

In the last years, different studies on N metabolism have improved our understanding of how diatom cells use N for growth [25,31–33]. Environmental ${\mathrm{NO}}_{3}^{-}$ uptake in diatoms mostly occurs through high-affinity nitrate transporters, diNRT2. However, little is known about mechanisms of ${\mathrm{NO}}_{3}^{-}$ signalling, including uptake and distribution among different cellular compartments.

In this study, we report the first characterization of the NPF family members in diatoms (diNPFs) through a multilevel approach which integrated data coming from fully sequenced diatom genomes [34] with the *TARA* Oceans datasets [35–37], that provide a unique combination of environmental, metagenomic and metatranscriptomic data, and the Marine Microbial Eukaryotic Transcriptome Sequencing Project (MMETSP) dataset, which provides over 650 protist transcriptomes [38].

These data, in combination with phylogenetic and expression analyses and structural predictions, uncover an unexpected evolutionary complexity of NPF transporters and offer the foundation for the genetic dissection of the extra- and intracellular transport system in diatoms, revealing once again the chimeric nature and the complex physiology of these successful microalgae.

## 2. Materials and methods

### 2.1. diNPFs identification in public genomes, *TARA* Oceans and MMETPS databases

An initial search of the diNPF sequences was performed using sequences described by [34] as queries in the following databases: JGI Genome Portal (https://genome.jgi.doe.gov/portal/), Ensembl Protist (https://protists.ensembl.org/index.html) and PLAZA Diatoms 1.0 (https://bioinformatics.psb.ugent.be/plaza/versions/plaza_diatoms_01/). Subsequently, a BlastP with the retrieved diatom sequences was performed on the genome of *Arabidopsis thaliana* using TAIR (https://www.arabidopsis.org/). NPFs are known to show high inter-phylum homology, making Blast search an appropriate method to find NPFs in different species/phyla [5]. Therefore, we performed an extensive BlastP search for putative *diNPF* genes in the Marine Atlas of *TARA* Oceans Unigenes (MATOU) database [36,37,39] and the MMETSP database [38], using diNPF homologues found in public genomes (table 1) as queries, with in-house developed platforms (http://bioinfo.szn.it/tara-blast-server/). The *TARA* Oceans gene catalogue was downloaded at http://www.genoscope.cns.fr/tara/ and the MMETSP at https://www.imicrobe.us/#/search/mmetsp. Nucleotide sequences were six-frame translated at https://www.ebi.ac.uk/Tools/st/emboss_transeq/. Each *TARA* Oceans Unigene is made by assembling individual cDNA reads using a 95% sequence identity clusterization [36].

**Table 1.** List and properties of diNPFs, modified and integrated from Rogato *et al*. [34]. Superscript letters in the protein ID column denote allelic pairs. The FcNPF256377 sequence was incomplete and was not analysed. TMs: transmembrane domains.

diatom	protein ID	chromosomal location	no. introns	AA length	no. TMs	top blastP hit in *A. thaliana* *(TAIR)*	% identity	genome reference
*P. tricornutum*	47 148	chr_12: 617 225–619 552 (−)	0	775	12	AtNPF8.2 AT5G01180.2	30	(Bowler *et al*. [27])
	47 218	chr_12: 843 193–845 146 (+)	0	650	12	AtNPF8.5 AT1G62200.1	27	
*T. pseudonana*	4104	chr_4: 182 289–184 968 (+)	3	765	12	AtNPF8.4 AT2G02020.1	28	(Armbrust [26])
	269 333	chr8: 269 367–271 586 (+)	3	592	12	AtNPF8.4 AT2G02020.1	25	
*T. oceanica*	32 021	scaffold_5551: 489–2290 (−)	2	547	12	AtNPF8.2 AT5G01180.2	25	(Lommer *et al*. [40])
	14 591	scaffold_23330: 1–700 (−)	0	233	5	AtNPF8.2 AT5G01180.1	29	
*P. multiseries*	190 665	scaffold_103: 217 004–220 392 (+)	5	688	12	AtNPF8.5 AT1G62200.1	42	
	226 109	scaffold_1727: 1943–4182 (−)	2	619	11	AtNPF8.4 AT2G02020.2	24	
*P. multistriata*	7930	scaffold_112: 108 896–111 744 (+)	0	791	12	AtNPF8.3 AT2G02040.1	28	(Basu *et al*. [41])
	12 290	scaffold_124: 54 727–57 165 (−)	2	654	12	AtNPF8.5 AT1G62200.1	27	
*F. cylindrus*	186 175^a^	scaffold_6: 1 219 622–1 221 532 (+)	2	565	12	AtNPF8.2 AT5G01180.2	25	(Mock *et al*. [42])
	204 239^a^	scaffold_110: 45 530–47 443 (+)	2	566	12	AtNPF8.2 AT5G01180.2	26	
	136 520^b^	scaffold_9: 1 493 580–1 495 600 (−)	2	571	12	AtNPF8.4 AT2G02020.2	24	
	256 377^b^	scaffold_75: 193 898–195 223 (−)	—	441	—	AtNPF8.1 AT3G54140.2	30	
	171 976	scaffold_11: 1 319 758–1 322 441 (−)	2	589	12	AtNPF8.4 AT2G02020.1	26	
	147 192	scaffold_15: 1 337 290–1 339 086 (+)	2	527	12	AtNPF8.1 AT3G54140.2	25	
	200 740	scaffold_56: 206 598–208 532 (+)	2	585	12	AtNPF8.1 AT3G54140.2	25	
*F. solaris*	15 278^c^	scaffold_89: 119 806–123 050 (+)	1	552	12	AtNPF8.4 AT2G02020.1	29	(Tanaka *et al*. [43])
	26 459^c^	scaffold_ 246: 21 487–25 295 (−)	1	561	12	AtNPF8.3 AT2G02040.1	27	
	17 535^d^	scaffold_118: 68 724–71 015 (−)	0	614	12	AtNPF8.4 AT2G02020.1	26	
	17 972^d^	scaffold_123: 425 437–427 851 (−)	0	684	12	AtNPF8.4 AT2G02020.1	27	
	20 187^e^	scaffold_144: 44 917–46 935 (−)	0	609	12	AtNPF5.14 AT1G72120.1	27	
	25 493^e^	scaffold_225: 74 815–76 805 (−)	0	609	12	AtNPF8.4 AT2G02020.1	24	
*C. cryptica*	26 601	chr83 : 3655–5942 (−)	1	737	12	AtNPF8.1 AT3G54140.2	29	(Traller *et al*. [44])
	35 672	chr114 : 10 006–12 057 (+)	2	623	12	AtNPF8.4 AT2G02020.1	25	
*S. robusta*	245 800	chr1142: 15 208–17 268 (+)	0	686	12	AtNPF8.4 AT2G02020.1	25	(Osuna-Cruz *et al*. [45])
	2800	chr3: 245 400–247 466 (−)	0	688	12	AtNPF8.4 AT2G02020.1	28	
	337 320	chr2795: 9912–11 528 (−)	0	538	11	AtNPF8.2 AT5G01180.2	27	
	22 590	chr35: 134 056–135 699 (−)	0	547	12	AtNPF8.5 AT1G62200.1	29	
	193 560	chr727: 5736–8118 (+)	1	748	12	AtNPF8.1 AT3G54140.2	23	
	262 280	chr1319: 16 210–18 853 (+)	1	849	12	AtNPF8.4 AT2G02020.2	33	

### 2.2. diNPFs alignment

Multiple sequence alignment was performed by subsequent rounds using the MAFFT v. 7 software at the MAFFT web portal (https://mafft.cbrc.jp/alignment/server/large.html). Initially, the experimental algorithm for a large number of short and similar sequences was used. At each alignment round, sequences not satisfying specific conditions (length <75 AA, similarity with reference genes 75%, quality of the sequence) were deleted from the alignment. In the second step, sequence alignments were performed at the MAFFT v. 7 web portal using standard options. TrimAl (http://trimal.cgenomics.org/) was used to remove spurious sequences or poorly aligned regions from the alignment [46]. Finally, the final alignment was obtained in-house using the ClustalW algorithm as implemented in Bioedit V. 7.0.5.3. Alignment statistics were calculated using MEGA v. 10.0.5 [47].

### 2.3. Phylogenetic analyses

In order to construct a robust multi-kingdom NPFs alignment for phylogenetic purposes, NPF sequences from bacteria, fungi, protists, plants and animal species retrieved from public databases were added to the initial alignment (the complete set of non-MATOU sequences is presented in electronic supplementary material, file S1A and B).

The best protein fitting model (BPFM) for the given alignment was searched using ProtTest as implemented in MEGA v. 10.0.5. The selected BPFM was LG + *γ* + I + F, where *γ* = 1.27. Maximum-likelihood phylogeny was inferred using IQ-TREE v. 1.6.12 for Windows system, with 1000 BS replicates. Bayesian inference was performed using MrBayes-3.2.7 for Windows system, using 2 chains; Bayesian inference required 28 000 000 generations to reach an average standard deviation of split frequencies <0.01.

### 2.4. Global meta-omics of MATOU diNPFs

All analyses in this section were performed in R v. 3.4.4-win. *Pearson correlation* and relative *p-*values were calculated in the R package ggplot2. Richness was calculated on unique *TARA* Oceans unigenes.

### 2.5. Culture conditions

Axenic strains of *Phaeodactylum tricornutum* Bohlin, for ecotypes Pt1 (CCMP632) and Pt4 (CCMP2559) [48,49], were obtained from the Provasoli-Guillard National Centre for Culture of Marine Phytoplankton. Cultures were grown in F/2 -Si medium [50] at 18°C under white fluorescent lights (90 µmol m^−2^ s^−1^), in a 12 h light/12 h dark photoperiod. In the experiments with *P. tricornutum*, the Pt1 ecotype grown in F/2 -Si medium [50] (882 µM NaNO_3_ as N source and a pH of 8.0) was used as control sample for the gene expression analysis. In the different experiments reported, cells were grown in F/2 medium containing 50 µM NaNO_3_, 1 mM NH_4_NO_3_ or 1 mM NH_4_Cl as N sources, and harvested after 5 days, 2 h after the onset of light. In addition, Pt1 cells were collected 1 h before and 2 h after the onset of light. Cells were grown respectively at pH 7.0, 8.0 and 9.0 and harvested after 5 days. In all the experiments, cells in the exponential phase of growth have been used.

### 2.6. RNA extraction and gene expression analysis

RNA extraction and quantitative real-time reverse-transcription polymerase chain reaction PCR (qRT-PCR) were performed as described in [51].

In detail, total RNA was isolated from 10^8^ cells using 1.5 ml TRI Reagent (SIGMA Life Science) according to the manufacturer's instructions, RNA concentration was determined using a NANODROP (ND 1000 Spectrophotometer) and qualitatively estimated by gel electrophoresis (1% agarose w/v). Two hundred nanograms of total extracted RNA was reverse transcribed with QuantiTect Reverse Transcription Kit (Qiagen) according to the manufacturer's instructions, and the qPCR reaction was performed according to [52].

1 µl of a 1 : 2 dilution of cDNA was used as template to amplify the *PtNPF* transcripts using 0.4 µM final concentration of the following primers: *PtNPF1* (ID 47148) with the primers: Pt47148_fw 5′- TTACGTGATTGGCTTGTCC -3′, Pt47148_rv 5′- GGTCCGGCGTTATTAACAGA -3′, and *PtNPF2* (ID 47218) with the primers: Pt47218_fw 5′- CTACGAAGTCGCCTTTACCG -3′, Pt47218_rv 5′- ATCTTCCAACCGCGTGATAC -3′. Primers were designed with Primer3 (http://primer3.ut.ee/). *RPS* (Ribosomal protein small subunit 30S; ID 10847) was used as the reference gene [52]. Real-time PCR amplification was performed using Light Cycler™ 480 SYBR GREEN I Master 2X (Roche) in a final volume of 10 µl. Each reaction was tripled for each gene in each sample using 384-well plates (BioRad) in the ViiA 7 Real-Time PCR System (Thermo Fischer). Thermocycler settings were 95°C for 10 min, 40 cycles at 95°C for 1 s and 60°C for 20 s, a gradient from 60°C to 95°C for 15 min, to finish with 5 min at 72°C. Data obtained were manipulated with the ViiA 7 Real-Time PCR system software. Fold-changes were obtained with the Relative Expression Software Tool-Multiple Condition Solver (REST-MCS) [53].

### 2.7. *In silico* analysis of the *diNPFs* non-coding sequences

The 5′-flanking regions (500–1000 bp region between the coding sequence of the gene of interest and the upstream gene) of *diNPF* genes for all diatom species whose genome is available were scanned by use of the MEME Suite program (v. 5.1.1) (http://meme-suite.org/tools/meme) [54], with a *p*-value cut-off of 10^−3^. The TOMTOM (v. 5.1.1) (http://meme-suite.org/tools/tomtom) tool, with default parameters, was used to assess motif occurrences in the analysed sequences and searched against the Eukaryotic DNA, JASPAR CORE and UniPROBE Mouse database. FIMO (v. 5.1.1) (http://meme-suite.org/tools/fimo) tool was used to research specifically two motifs previously identified by [33].

### 2.8. Structural prediction of diNPFs

Structural models of diNPFs were built by homology using the coordinates from the crystal structures of one plant NPF or bacterial POTs as templates. Homology models were obtained using the Swiss-Model Workspace [55,56]. PyMol was used for molecular visualization (The PyMOL Molecular Graphics System, Schrödinger, LLC).

### 2.9. Prediction of sub-cellular localization

The sub-cellular localization was predicted by running diNPF sequences through the LocTree3 software as reported in [19] (https://rostlab.org/services/loctree3/) [57], accessed through the PredictProtein service [58]. Obtained results were compared with other software, such as WolfPSort (https://wolfpsort.hgc.jp/). The sub-cellular localization for the full-length sequences was also predicted through the SignalP (http://www.cbs.dtu.dk/services/SignalP/) software that uses an algorithm based on signal peptide research.

## 3. Results

### 3.1. *diNPF* genes in diatom genomes, in *TARA* Oceans and in MMETPS databases

We looked for diNPFs in all diatoms with complete genomic sequences [34,40,41,43–45], expanding previous reports. In agreement with what previously reported in [34], we found that diatoms possess two genes encoding putative NPFs. Notably, *Fragilariopsis cylindrus* [42]*, Fistulifera solaris* [43] and *Seminavis robusta* [45] contain more than two copies, possibly due to the existence of divergent alleles, allopolyploidy and extensive gene duplication phenomena described respectively in the three species.

Specific features of the identified sequences are reported in table 1. The NPF plant families have been sub-classified in eight phylogenetic clades, interestingly all the identified diNPF sequences share the highest level of homology (between 22% and 42%) with *A. thaliana* NPFs belonging to Clade 8 that include five proteins characterized as di/tri-peptide transporters and localized on the plasma membrane or on the vacuole membrane [5].

To expand the repertoire of NPF diatom sequences, BlastP searches were also made against the MMETSP database and the *TARA* Oceans database [36], retrieving 245 (191 belonging to diatom species) and 42 sequences, respectively.

### 3.2. diNPFs phylogeny

To elucidate the molecular evolution and phylogenetic relationships among the NPF proteins, we constructed a phylogenetic tree including the diNPF sequences identified from available sequenced genomes, the *TARA* Oceans gene atlas, and the MMETSP dataset. The diNPFs alignment (electronic supplementary material, file S2) consists of 448 sequences with a length of 271 aa (including gaps), including sequences from five out the six biological kingdoms, with only Archaea being excluded. Of the 448 sequences, 20 belonged to bacteria, 6 to fungi, 344 to protists (266 of which to diatoms), 71 to plants and 7 to animals.

**Figure 1.**
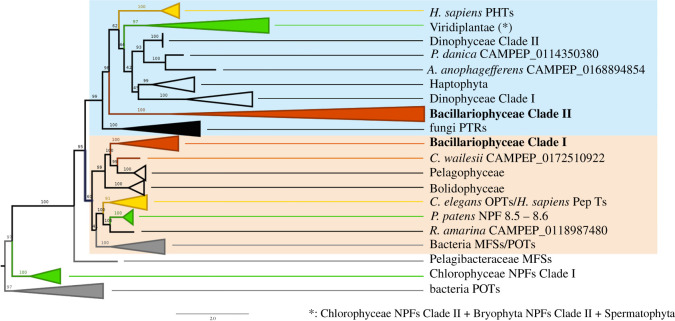
NPFs evolutionary relationships inferred using the maximum-likelihood and Bayesian inference approaches. Numbers over the nodes represent bootstrap values. For all bootstrap values greater than 75, a posterior probability greater than 0.75 was also found. Branches were collapsed at high taxonomical levels. Cyan box indicates the NPFs Clade II; salmon box indicates the NPFs Clade I. Red branches are used for diNPFs Clades I and II, yellow for Metazoa, green for plants and green algae, grey for bacteria.

Phylogenetic relationships between sequences are shown in figure 1 and electronic supplementary material, figure S1, and described in electronic supplementary material, file S3. Bacterial POTs are used as an outgroup to all the other sequences. Two Chlorophyceae NPFs belong to a sister group (Chlorophyceae Clade I) to all the other ingroup genes, suggesting that these two genes derive from a relatively recent event of lateral gene transfer (LGT). A single bacterial gene from *Pelagibacteraceae bacterium* (MFS transporter) is basal to the two main NPFs clades (NPFs Clade I and Clade II), with NPFs Clade I including genes belonging to species from four biological kingdoms (Prokaryota, Protista, Plantae and Animalia). Interestingly, Viridiplantae are herein however only represented by two genes from the common moss *Physcomitrella patens*, while no Streptophyta and no Chlorophyceae are included in this clade. In diatoms Clade I (electronic supplementary material, file S4A, B), only two sequences are from genes expressed in the wild (*TARA* Oceans MATOU). No bacterial NPFs are found in the NPFs Clade II, while all fungal NPF sequences belong to this clade. Herein, the diatoms Clade II (electronic supplementary material, file S4A, B) is a sister clade to all other Protista, Plantae and Animalia NPFs. This clade includes 200 diatom sequences, of which 40 (out of a total of 42) are expressed in the wild. The main subclade within NPFs Clade II includes two Dinophyceae and one Haptophyta clades, plus one sequence from *Aureococcus anophagefferens* (Pelagophyceae) and one from *Pteridomonas danica* (Dictyochophyceae). Green plants are herein represented by sequences from Chlorophyceae, Bryophyta and Streptophyta.

An in-depth analysis of the evolutionary relationships of the diNPFs is shown in electronic supplementary material, figure S1 and File S4C. diNPFs included in the Clade I diNPFs are poorly expressed in the global ocean, with only 2, out of 42, MATOU diNPFs being found in this clade (namely, MATOU-v1_19401141 belonging to the *Pseudo-nitzschia* genus and MATOU-v1_113685264 to *Fragilariopsis kerguelensis*). Moreover, there are a number of species which appear to have sequences only in Clade I and not in Clade II (namely, *Cyclophora tenuis*, *Dactyliosolen fragilissimus*, *Entomoneis *sp.**, *Grammatophora oceanica*, *Leptocylindrus* genus, *Odontella *sp.**, *Proboscia* genus, *Striatella unipunctata*), while an even bigger number of species/genera seem to have sequences only in diatoms Clade II (e.g. *Skeletonema* genus and *Thalassiosira* genus).

Albeit the focus of the present study is on diatoms, our phylogeny elucidates evolutionary relationships also in other phytoplankton. Interestingly, no other phytoplankton group shows the same two-clade dichotomy of diatoms: Pelagophyceae and Bolidophyceae are exclusively found in the NPFs Clade I, while Dinophyceae and Haptophyta are all exclusively found in the NPFs Clade II (where Dinophyceae show two well-separated subclades). The only exception is given by the Dictyochophyceae, which are present in both NPFs Clade I and II, although with a single sequence in each clade.

A puzzling observation is the absence of NPFs sequences in *Chaetoceros,* one of the largest genera of diatoms, with many abundant and cosmopolitan species [59]. Busseni *et al*. [19] had identified high-affinity ${\mathrm{NO}}_{3}^{-}$ (NRT2) and ${\mathrm{NH}}_{4}^{+}$(AMT) transporters for this genus, while, with the exception of one single sequence for *Chaetoceros sp*. (CAMPEP_0176481300), we were unable to detect any NPFs for *Chaetoceros* in the same datasets*,* including the transcriptome of *Chaetoceros decipiens* [60].

Diatom Clade I and Clade II *NPFs* are *bona fide* distinct genes, while at the present stage it would require more in-depth studies to assert whether within-clade distinct species-specific lineages correspond to different genes or to isoforms of the same gene, mostly due to the fact that many *TARA* Oceans MATOU sequences tend to be partial/incomplete. Nonetheless, the above results are in line with those reported for diatom genomes in the previous section.

### 3.3. *diNPF* distribution in the global ocean

**Figure 2.**
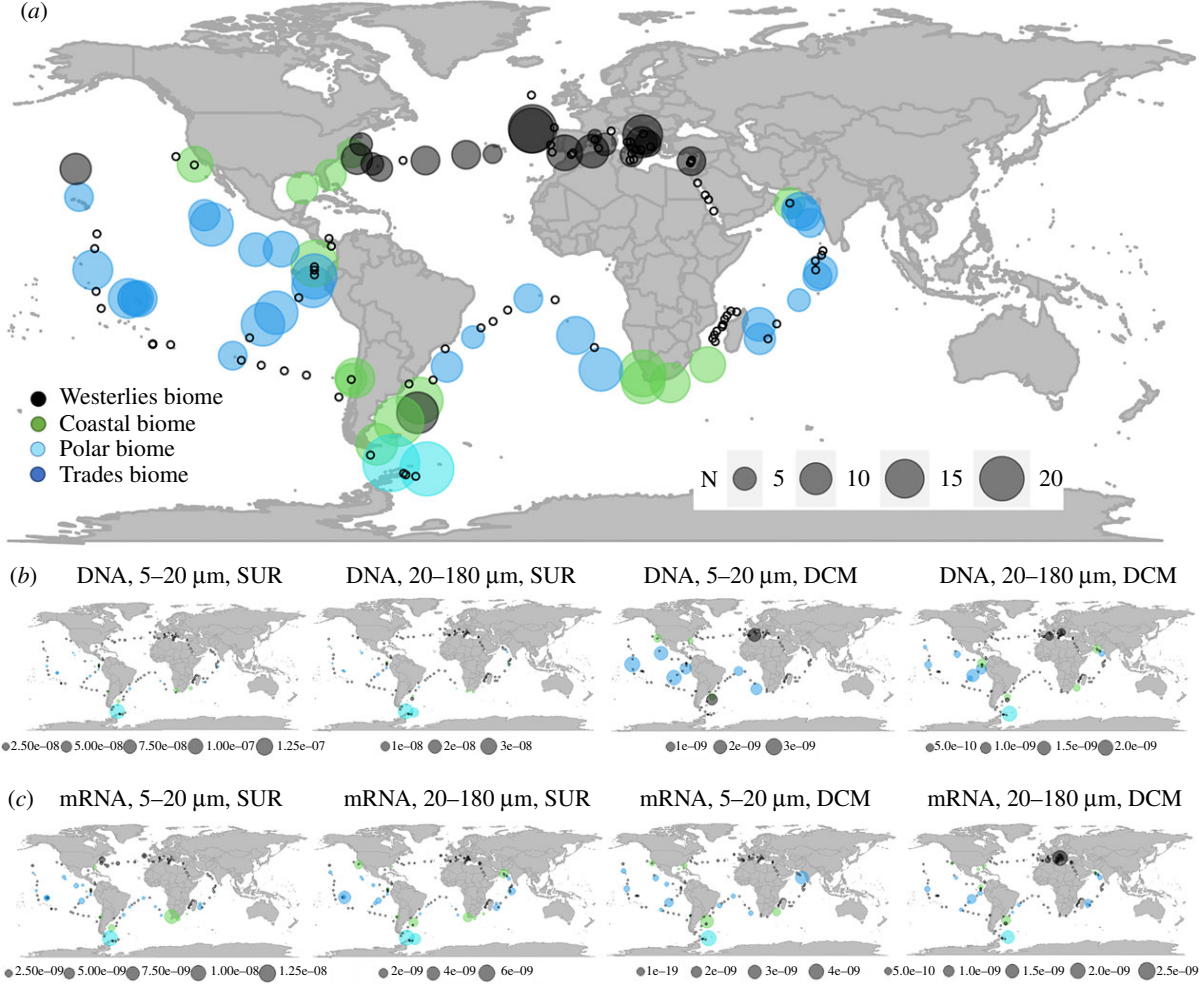
*diNPF* richness (*a*), and *diNPF* DNA (*b*) and mRNA (*c*) relative abundance in the global ocean. Data mapped are from the *TARA* Oceans dataset. Sampling stations are coloured according to the belonging biome. White dots indicate *TARA* Oceans stations where no NPFs were present. Circle size is proportional to abundances. In (*b*) and (*c*), data were mapped by size classes and sampling depth. SUR, surface, DCM, deep chlorophyll maximum.

We analysed *diNPF* richness, by means of a mere count of distinct diNPFs found in the *TARA* Oceans eukaryote unigene catalogue [19,36]. diNPFs richness (figure 2*a*) is high in the South Polar region and in the Coastal biome of the Southern hemisphere. Other peaks in *diNPF* richness are found in the Adriatic Sea, in the North Atlantic Sea and in the Pacific Ocean along with the Equatorial upwelling. Conversely, the middle North Atlantic Ocean, the Western Mediterranean basin, and some stations in the Indian Ocean show a minimum in the number of distinct *diNPFs* detected.

*diNPF* DNA relative abundance is shown in figure 2*b*. Data are separately presented for two size classes (5–20 µm and 20–180 µm [19]) and for two different sampling depths: surface (SUR, around 3–5 m depth) and deep chlorophyll maximum (DCM, tens of metres below surface). *diNPFs* are usually more abundant at the DCM than at SUR, and very abundant at the South Pole, with the exception of *diNPFs* of the 5–20 µm size class, at the DCM. Conversely, the comparison of *diNPF* mRNA occurrences (figure 2*c*) shows more similarity between different size classes and depth than *diNPF* DNA. This is of note, since low-DNA-high-mRNA (and vice versa) levels in a given sampling station are the only proper way to infer patterns of overexpression (and downregulation) of genes in the *TARA* Oceans dataset [61,62]. As an example, the high-DNA-high-mRNA level detected in the South Polar sampling stations (fraction 5–20 µM, SUR, and fraction 20–180 µM, DCM and SUR) is representative of basal expression of diNPFs, while low-DNA-high-mRNA level (South Pole, fraction 5–20 µM, DCM) is likely representative of few, highly expressed diNPFs. The comparison of DNA and mRNA *diNPF* levels is in general suggestive of highly expressed genes at the surface, and of low expression of the same genes at the DCM (with exceptions). *diNPFs* are likely to be highly expressed in superficial waters in the region of the Equatorial upwelling in the two size classes.

**Figure 3.**
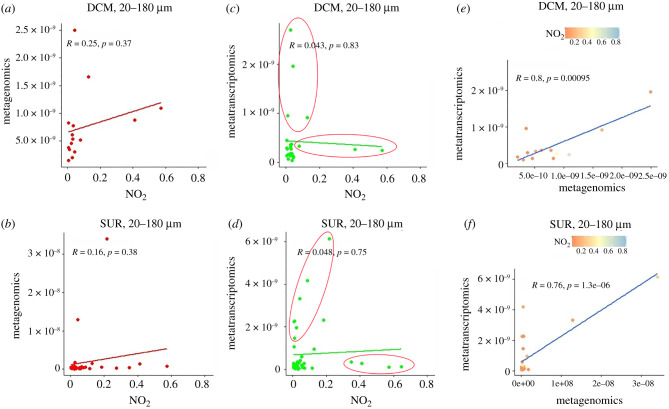
Correlation by means of Pearson's *r* of *diNPF* DNA (Metagenomics, (*a*,*b*)) and mRNA (Metatranscriptomics, (*c*,*d*)) with NO_2_ levels in the global ocean for the size class 20–180 µm, at both DCM and SUR sampling depths. In both cases, DNA and mRNA levels have been correlated one to the other and in relation to NO_2_ level (*e*,*f*). Red circles in the mRNA versus NO_2_ scatter plots indicate type I and type II responses.

Under the same, above-mentioned assumption on the way to detect transcriptional variations in the oceanic samples, *diNPF* DNA and mRNA levels in the *TARA* Oceans sampling stations were correlated with eco-physiological variables. In most cases, *diNPF* DNA and mRNA levels showed very similar trends when correlated with the considered variables (electronic supplementary material, figures S2, S3 and S4). This was also true when *diNPF* DNA and mRNA levels were correlated with NO_2_ and NO_3_ environmental concentration (electronic supplementary material, figure S2A). However, when *diNPF* DNA and mRNA levels were correlated with NO_2_ oceanic concentrations, in two cases (namely, 20–180 µm, at both SUR and DCM sampling depths), mRNA levels showed trends indicative of two types of response (figure 3). In the first type, no variation in the mRNA levels are detected in increasing NO_2_ environmental concentration. In the second type of response, mRNA levels are increasing in constant or slightly increased NO_2_ environmental concentration (figure 3). Type I response is suggestive of long-term adaptation to NO_2_ in the surrounding seawater, while type II response is more likely linked to short-term acclimation to NO_2_ concentration.

**Figure 4.**
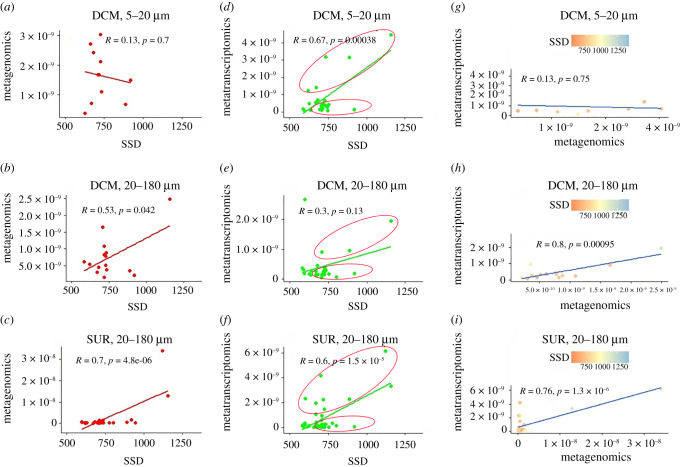
Correlation by means of Pearson's *r* of *diNPF* DNA (Metagenomics (*a*,*b*,*c*)) and mRNA (Metatranscriptomics (*d*,*e*,*f*)) with sunshine duration (SSD) levels in the global ocean for the size class 20–180 µm, at both DCM and SUR sampling depths. In both cases, DNA and mRNA levels have been correlated one to the other and in relation to SSD level (*g*,*h*,*i*). Red circles in the mRNA versus SSD scatter plots indicate type I and type II responses.

Interestingly, the two types of response are also detected when *diNPF* DNA and mRNA levels are correlated with sunshine duration (SSD) (figure 4). Herein, type I and type II responses in mRNA levels are detected at the DCM (both size classes) and at SUR (20–180 µm). These results suggest that the response of *diNPFs* to sunshine duration is rather complex, with different communities responding differently.

### 3.4. Expression patterns of *diNPFs* in *Phaeodactylum tricornutum*

**Figure 5.**
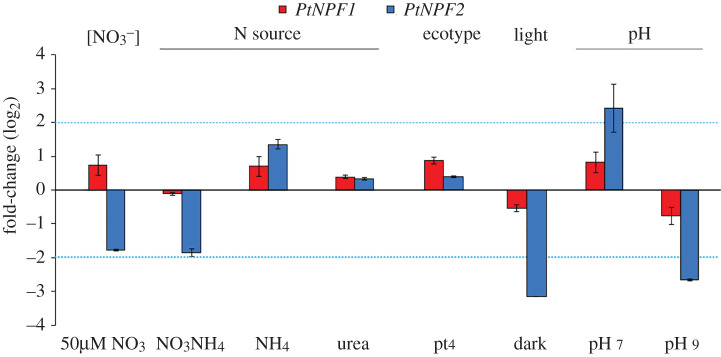
Expression profile analysis of *PtNPFs* genes as assessed by qPCR in different N concentrations, in different N sources, in different ecotypes, in light or dark condition and at different pHs. Experiments were performed separately, using as control *P. tricornutum* wt Pt1, grown in 882 µM ${\mathrm{NO}}_{3}^{-}$ at pH 8, collected during the light phase. Red bars: logFC (fold-changes) for *PtNPF1*; blue bars: logFC (fold-changes) for *PtNPF2*. Black bars represent standard deviations.

We performed an exploration of the expression patterns of *diNPF* genes to integrate data from *TARA* Oceans meta-omics analyses, surveying independent gene expression studies already reported in the literature (electronic supplementary material, table S1). Furthermore, to determine the regulation of *diNPFs* in response to continuously changing environmental factors, we analysed the gene expression pattern of the two *P. tricornutum NPFs* genes, *PtNPF1* (ID47148) *and PtNPF2* (ID47218), in wild-type (wt) Pt1 cells, grown under different culture conditions (figure 5).

First, to investigate whether *diNPF* genes can respond to diverse N concentrations and sources, we analysed the expression of the genes in wt cells grown under N starvation (50 µM NO_3_), and exposed to different N sources (1 mM NH_4_Cl, 1 mM NH_4_NO_3_ or 1 mM urea) and we observed that the expression profiles of the genes were not altered. Ten ecotypes of *P. tricornutum* from different ecological niches, from sub-polar to tropical latitudes, have been identified and characterized [48,49]. We analysed expression levels in ecotype Pt4, known to have adapted to high latitude environmental conditions [48]. No differential expression was observed between the two ecotypes Pt1 and Pt4 (figure 5).

Interestingly, a regulation of the *diNPFs* transcripts in dark was previously observed in different species [42,63,64] (electronic supplementary material, table S1). Therefore, we tested the expression of the two *PtNPFs* genes in Pt1 cells collected during the dark period (12 h), observing a clear decrease of the *PtNPF2* mRNAs while *PtNPF1* was only slightly less transcribed (figure 5), suggesting a diurnal regulation of *PtNPF2*.

Finally, since previous reports indicated a possible relation with pH [65], we tested regulation under different pH values and revealed that *PtNPF2* expression was repressed at alkaline pH, while it was induced at pH 7 as compared to growth at pH 8 (figure 5).

### 3.5. Putative transcription factor binding sites in the *diNPF* genes upstream regions

With the aim of identifying possible regulatory motifs involved in the regulation of the *diNPFs* transcription, we scanned the 5′-flanking regions using the MEME Suite program (Multiple Expression motifs for Motif Elicitation) [54] a tool for identification, analysis and comparison of sequence motifs. At the time of the study, only for *P. tricornutum, P. multistriata, T. pseudonana* and *F. cylindrus*, it was possible to identify the intergenic region of about 500–1000 bp upstream of *diNPF* coding sequences, which was the input for the program. Interestingly, three motifs are present in all the sequences (electronic supplementary material, figure S5). We compared these motifs using TOMTOM to the JASPAR CORE database, a curated set of eukaryotic TFBSs (Transcription Factor Binding Sites), to identify the class of TFs that might bind and regulate this site. This returned five motifs that bind TFs found in other organisms as plants or fungi through a zinc finger domain. Two other motifs were identified in many but not all upstream *diNPF* sequences. In particular, the most represented of these (the red one in electronic supplementary material, figure S5), returned 11 motifs when compared with a Eukaryotic motifs database using TOMTOM.

Most of these motifs were bound by plant TFs involved in the regulation of gene expression by environmental or stress factors. In particular, seven plant TFs models belong mainly to the ethylene-responsive transcription factors (ERF) class, which regulate transcription in response to hormone levels like ethylene, abscisic acid or IAA (indole-3-acetic acid).

Finally, the FIMO tool was used to verify the presence of two nitrate regulated motifs, HNS_A and HNS_B, previously identified by [33] in upstream *diNPF* sequences. These motifs were identified only upstream of **FcNPF136520*, PtNPF1* and *PtNPF2* (*p*-value > 10^−5^).

### 3.6. Structural modelling of diNPFs

Structural data are available for one plant NPF, *A. thaliana* NPF6.3 [12,14] and several of the evolutionary related bacterial POTs [6,11,13,66]. Based on sequence alignment results between diNPFs and the sequences of NPFs/POTs of known structure, we picked different templates for model building. For Clade I diNPFs, the top-ranked template was the bacteria *Shewanella oneidensis* peptide transporter PepTso (pdb: 4uvm) [67]; for Clade II diNPFs, it was the plant *A. thaliana* NPF6.3 (pdb: 4oh3) [14]. We report structural models for *PtNPF1* and *PtNPF2* from *P. tricornutum* as representatives of Clade II and Clade I diNPFs, respectively. Structure homology models were obtained using the SWISS-MODEL workspace [55,56].

**Figure 6.**
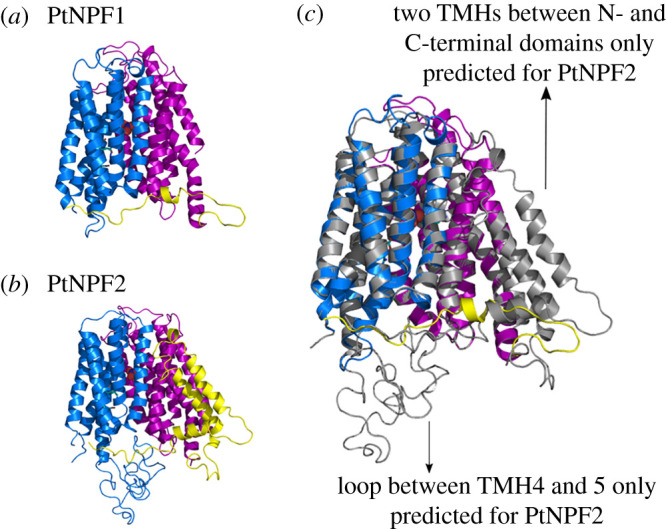
Separated and overlapped structural models of *P. tricornutum* PtNPF1 (Clade II) and PtNPF2 (Clade I). (*a*) The PtNPF1 model (N-terminal in blue, C-terminal in magenta and lateral helix in yellow) was obtained using the crystal structure of the *A. thaliana* NPF6.3 (pdb: 4oh3) as template [14]. (*b*) The PtNPF2 model was obtained using both the AtNPF6.3 and the *S. oneidensis* peptide transporter PepTso (pdb: 4uvm) [67] as template structures. (*c*) The two structural models overlapped to highlight their structural differences between diNPF belonging to two different clades.

Complete diNPF sequences belonging to the two Clades are predicted to fold into 12 TMHs organized in two bundles, the N- and the C-terminal domains (figure 6). However, our structural models together with multiple sequence alignment revealed differences in the length and position of the loops between TMHs. First, all Clade I diNPFs have insert sequences of variable lengths between TMH4 and 5, while none of the Clade II diNPFs has an insert sequence in this position (table 2). Second, both Clade I and II diNPFs contain an insert sequence between TMH6 and TMH7 (table 2). In Clade II diNPFs this insert is predicted to fold as a later helix like in plant NPFs, while in Clade I diNPFs, it is predicted to fold into a later helix and into two TMHs as observed in the crystal structure of bacterial POTs. There is not a known role for these additional TMHs, but since they are absent in fungal, mammalian and plant transporters, it has been suggested they contribute to stability or folding more than to the transport mechanism [15].

**Table 2.** Properties of PtNPFs and templates used to build their structural homology models.

clade	representative protein	length (aa)	structural elements	Pdb of templates
Clade I	*P. tricornutum* NPF2 (ID 47218)	650	long loop between TMH4–5 later Helix + 2 TMH between TMH6–7	4uvm
Clade II	*P. tricornutum* NPF1(ID 47148)	775	later helix	4oh3

**Figure 7.**
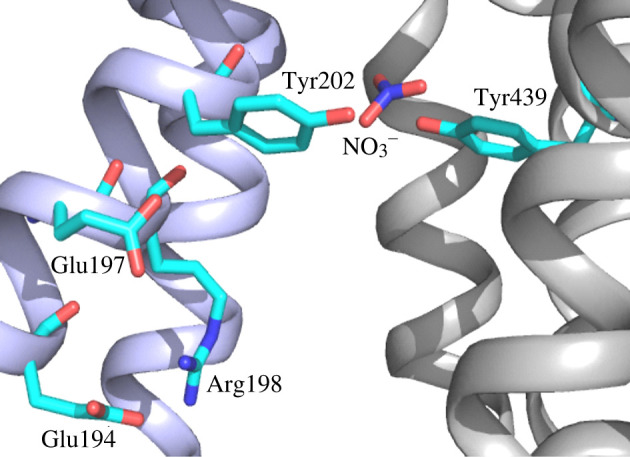
Zoomed in view of the putative ${\mathrm{NO}}_{3}^{-}$ binding site of PtNPF1. Residues Glu194, Glu197 and Arg198 belong to the conserved ExxER motif. Residues Tyr202 and Tyr439 form part of the substrate binding site. TMHs belonging to the N- and C-terminal domains are in light blue (left) or light grey (right), respectively.

**Figure 8.**
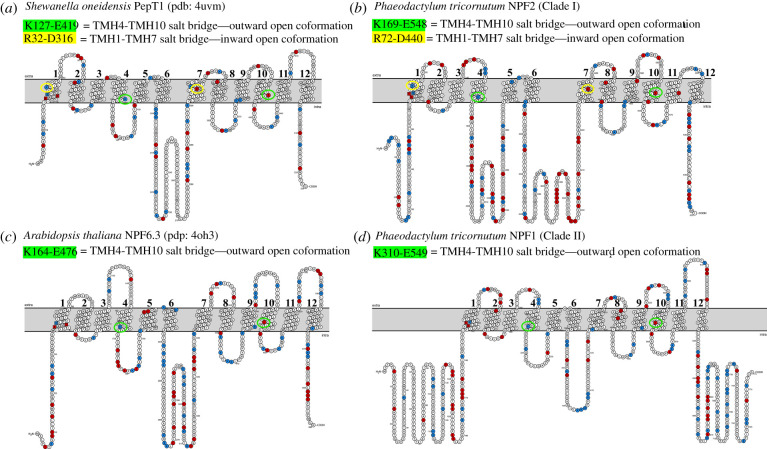
Visualization of the transmembrane topology of bacterial, plant and diatom POTs/NPFs. Topology plots showing the 12 transmembrane portions of (*a*) *Shewanella oneidensis* PepT1, based on its crystal structure (pdb: 4uvm) [67]; (*b*) *P. tricornutum* NPF47218 (PtNPF2), based on its structural model; (*c*) *Arabidopsis thaliana* NPT6.3, based on its crystal structure (pdb: 4oh3) [14]; (*d*) *P. tricornutum* NPF47148 (PtNPF1), based on its structural model. ExxER motif amino acids are indicated by squares instead of circles; arginine/lysine in blue; glutamic acid/aspartic acid in red. Residues forming a salt bridge between TMH1 and TMH7 or TMH4 and TMH10 are enclosed within a yellow or green circle, respectively. Figure created with the Protter web application (http://wlab.ethz.ch/protter).

Sequence alignment and structural models of diNPFs allowed us to identify key amino acids involved in proton or substrate binding and transport (table 2 and figures 7 and 8). First, we looked at the ExxER motif which has an important role in coupling proton binding to peptide or ${\mathrm{NO}}_{3}^{-}$ transport. In the crystal structures from bacterial POTs and one plant NPF, the motif is located on TMH1 with the chargeable amino acids available for proton binding in the substrate cavity. In diNPFs, the chargeable amino acids of the ExxER motif are strictly conserved and our structural models confirm that the chargeable residues are correctly oriented in the access cavity (table 2 and figures 7 and 8).

Salt bridges have been identified in POTs and NPFs and have been implicated in orchestrating conformational changes of the transporters and contributing to the alternating-access mechanism [3,16,68]. One salt bridge between residues on TMH4 and TMH10 forms in the outward-open conformation [15,16]. These residues are conserved in all diNPFs (electronic supplementary material, table S2). A second salt bridge between TMH1 and TMH7 that is predicted to form in the inward open conformation in some bacterial POTs but not in plant NPFs (with the exception of two NPFs from a moss) is conserved in all Clade I diNPFs, but not in Clade II. Interestingly, Clade I diNPFs align with the two moss NPFs with residues that can form the TMH1–TMH7 salt bridge [69].

Then, we looked at residues involved in substrate binding. The ${\mathrm{NO}}_{3}^{-}$ binding site in the plant AtNPF6.3 crystal structure features a protonated histidine, His356 which forms an electrostatic interaction with the ${\mathrm{NO}}_{3}^{-}$ [12,14]. diNPFs have a conserved tyrosine in the corresponding position (figure 7; electronic supplementary material, table S2). Another tyrosine (or phenylalanine) from TMH1 is located in close proximity and may contribute to creating a hydrophobic pocket for the substrate (figure 7; electronic supplementary material, table S2).

### 3.7. Predicted sub-cellular localization

The sub-cellular localization of 325 diNPF sequences was predicted by exploiting the LocTree3 software via homology-based inference between proteins of known localization [57]. The reliability of the LocTree3 software was previously tested by [51], confirming the sub-localization of different plant sequences whose localization has been experimentally verified.

Sixty-six per cent of the diNPF sequences are predicted to be plasma membrane proteins (table 3). These predictions are based on sequence homology with (i) plant NPFs, mainly *A. thaliana* NPF8.1 and 8.2, that are involved di/tri-peptides transport; (ii) a *C. elegans* peptide transporter PEPT2, responsible of proton-dependent uptake of di/tri-peptides and (iii) two bacterial POTs, *E. coli* DtpD (YbgH) and DtpB (YhiP), which are proton-dependent transporters of di/tri-peptides.

**Table 3.** Predicted sub-cellular localization of diNPFs, including sequence number and corresponding percentage that are predicted to a specific sub-cellular localization, and homologous Eukaryota and bacteria proteins that drove these predictions, when available.

localization	N° Sequences	% sequences (%)	homologous proteins
plasma membrane	216 (52 Clade I and 164 Clade II)	66	PTR1 (NPF8.1)–PTR5 (NPF8.2) *Arabidopsis thaliana* PEPT2 *Caenorhabditis elegans* (peptide transporter) DtpD–DtpB *Escherichia coli* (peptide permease)
mitochondrial membrane	26 (7 Clade I and 19 Clade II)	8	reconstructed path by software
vacuole membrane	80 (80 only Clade II)	25	PTR2 (NPF8.3) A*rabidopsis thaliana* (Protein NRT1/PTR Family)
secreted	2 (2 only Clade II)	1	reconstructed path by software
endoplasmic reticulum	1 (1 only Clade II)	0	reconstructed path by software
total	325	100	

Interestingly, a significant percentage of sequences was predicted to be located at the vacuole membrane (25%) by homology with AtNPF8.3 (table 3). AtNPF8.3 is a proton-coupled, voltage-dependent di/tri-peptides transporter located at the vacuole and can recognize a variety of different amino acid combinations [70]. Furthermore, since bacterial POTs are essentially oligopeptide transporters, while plant NPFs are capable of carrying different substrates, including ${\mathrm{NO}}_{3}^{-}$ and other molecules, it is interesting that all diNPFs sequences predicted to the vacuole belong to the Clade II diNPFs.

Finally, 8% of sequences were predicted to the mitochondrial membrane (table 3).

## 4. Discussion

Diatoms are known to thrive in conditions that are less favourable for other phytoplankton, and this ability has often been linked to effective systems to deal with variable availability of nutrients, especially N and iron [71,72]. Several elegant studies are available on the N metabolism in diatoms [25,32,33]. Still, many questions remain open and the role of many components known to have major functions in other systems remains undefined. To transport ${\mathrm{NO}}_{3}^{-}$, diatoms mostly rely on high-affinity transporters, NRT2s [34], coherently with the need to deal with very low ${\mathrm{NO}}_{3}^{-}$ concentrations in seawater, and in principle, they would not need low-affinity transporters. However, diatoms do harbour low-affinity transporters that are related to bacterial POTs and plant NPFs. While in plants NPFs have expanded, diatoms mostly contain two NPFs. Our extensive survey of NPFs conservation in diatom genomes, transcriptomes, metagenomics and metatranscriptomics data indicate that the two NPFs homologues are broadly retained. Whether their role is ${\mathrm{NO}}_{3}^{-}$ transport, thereby contributing to diatoms ability to sense and efficiently uptake N, or in other as yet undefined functions, remains to be experimentally demonstrated. In this paper, we present several lines of evidence which provide working hypotheses.

Our evolutionary analysis shows that two distinct diNPFs clades each contained one of the two homologues for most of the species analysed, with some interesting exceptions whereby some species had two genes clustered together in a single clade and no gene in the other clade. This, together with the absence of NPFs in the genus *Chaetoceros*, is suggestive of an ongoing process of evolution of this family in diatoms. The specificity of the evolutionary processes in diNPFs is even more highlighted by the unicity of their two-clade dichotomy among the phytoplankton. In order to move forward in the biological interpretation of the role of *NPF* genes, future studies may benefit from extending analyses of other phytoplankton groups. As to why *Chaetoceros* represents an exception, the NPFs absence may be due to the presence of alternative genes with conserved function or to different adaptations for N transport. We also note that in environments with fluctuating N, *Chaetoceros* can preferentially transport NH^+^_4_ derived from N-fixing bacteria in the phycosphere [73].

The NPFs evolutionary relationships are complex, and at the present stage, it is not clear whether these are resulting from events of LGT, from an even more complex pattern of specific gene loss or from a mixture of the two. It is, however, of note that Clade I and Clade II NPFs differ in kingdom specific inclusion (Bacteria in Clade I, Fungi in Clade II) and that Viridiplantae are represented only by Bryophyta in Clade I, while Chlorophyceae and especially Streptophyta are present in Clade II.

Our structural analysis confirmed phylogenetic observations, revealing that Clade I diNPFs have structural features that are found in bacterial POTs, but not in plants, while Clade II diNPFs are structurally closer to plant NPFs. This specific evolutionary history may be reflected in functional specialization [61].

In recent years, several gene expression analyses were performed comparing diatom cells grown in different culture conditions. Among the conditions most widely studied is growth under different sources of nitrogen, from ${\mathrm{NO}}_{3}^{-}$ to ${\mathrm{NO}}_{2}^{-}$, ${\mathrm{NH}}_{4}^{+}$, NO_3_NH_4_ and urea [25,33], and different ${\mathrm{NO}}_{3}^{-}$ concentrations, to observe the regulation of gene expression in conditions of N starvation [25,33,71,74–77]. In line with our results (figure 5 and electronic supplementary material, figures S2 and S3), in almost none of these conditions the expression of *diNPFs* is reported to be regulated, with the exception of two *NPFs* of *F. cylindrus*, FcNPF147192 and FcNPF200740 [74] (electronic supplementary material, table S1).

The other main parameter often considered in diatom gene expression studies is light: there is evidence of a cross-talk between light and nutrient conditions, indeed the light-dark cycle is able to influence the nutrient uptake as well as nutrient redistribution inside the cell [78,79]. Despite no differential expression of *P. tricornutum NPFs* has been reported in the literature neither at different light wavelengths [80] nor at different light intensities [81], our meta-omics analyses of *diNPFs* expression profile in the ocean and comparison of DNA and mRNA *diNPF* levels are in general suggestive of highly expressed genes at the surface and equatorial regions, in which light intensities are particularly high (figure 2). Moreover, for the light–dark cycle, significant downregulation is reported in the darkness for *PtNPF2* [64], confirmed by our *PtNPF* genes expression analyses (figure 5), and for TpNPF4104 [63] (electronic supplementary material, table S1). It is of note that analyses of the *diNPFs* expression profile in the ocean showed two response patterns (one indicative of long-term adaptation, the other of short-term acclimation) to environmental SSD in specific sampling stations. Furthermore, in the cold-adapted *F. cylindrus*, where approximately 25% of the diploid genome consists of genetic loci with alleles that are highly divergent and probably involved in adaptation to environmental fluctuations in the Southern Ocean [42], different *diNPFs* behave differently in response to darkness: FcNPF204239, FcNPF256377, FcNPF171976 and FcNPF136520 are downregulated while FcNPF147192 and FcNPF200740 are upregulated [42]. This different regulation of the *FcNPFs* suggests that in *F. cylindrus* these genes have evolved differently to respond to different environmental conditions, promoting the adaptation of this species in the Southern Ocean extreme and variable environments, where daily and seasonal variations, the thickness of the pack and deep mixing result in a low average of light intensities and considerable periods of light limitation [82].

Intriguingly, we observed a pH effect in regulating the expression of the *PtNPF* genes, and in particular of *PtNPF2* (figure 5). This result is consistent with the results reported in [65] (electronic supplementary material, table S1), in which transcriptomic analyses in *T. pseudonana* showed the upregulation of TpNPF4104 at high pH.

**Figure 9.**
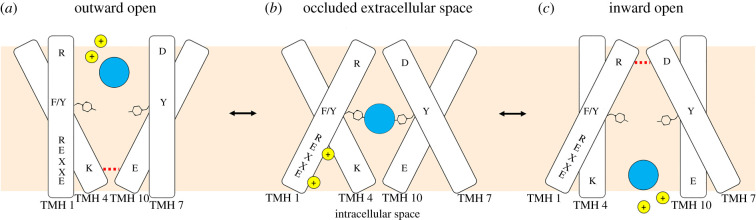
Proposed model for diNPFs functioning, with alternating-access mechanism. Dashed red lines represent salt bridges: the TMH4–TMH10 salt bridge is present in both diNPFs clades, while TMH1–TMH7 salt bridge is only present in Clade I diNPFs. Small yellow circles represent protons, while large blue circles represent a substrate, which could be NO^−^_3_ or other molecules.

Structural information allowed us to hypothesize a model of functioning of diNPFs, which would share the same alternating-access mechanism as plant NPFs and bacterial POTs [16,66] (figure 9): (i) the protein is in an open conformation towards the extracellular space stabilized by a salt bridge between TMH4 and TMH10, present in all diNPF sequences; (ii) once the substrate and protons bind to the transporter, the protein moves to an occluded conformation; (iii) the protein opens towards the intracellular space, protons and substrate are released in the cytoplasm. A new salt bridge may form between TMH1 and TMH7 in the inward open conformation. However, residues that form this bridge are only conserved in some bacterial POTs and in Clade I diNPFs (figure 8*a* and *b*), but not in Clade II diNPFs and plant NPFs—with the exception of two NPFs from a moss that cluster with Clade II diNPFs (figures 8*c*,*d* and 9).

In bacterial POTs and most plant NPFs, the residues responsible for proton binding and transport are two highly polar glutamate residues and the lysine residue typical of the ExxER motif located on TMH1, and for some POTs a glutamic acid found on TMH7, that is also responsible for the TMH1–TMH7 salt bridge [16,66,69]. Residues in the ExxER motif are conserved in diNPFs, suggesting sensitivity to the pH that can trigger conformational changes, confirming that the mechanism of substrate transport in coordination with proton transport is conserved in diNPFs.

Considering that the concentration of ${\mathrm{NO}}_{3}^{-}$ in the ocean is in the order of micromolar, the analysis of the *TARA* Oceans database and the gene expression profiles suggest that the expression of these genes is not strongly regulated by external ${\mathrm{NO}}_{3}^{-}$ availability, although we could detect a weak correlation with NO_2_ levels. Although the structure of the proteins has so far not been used to predict the substrate specificities of NPF transporters, and the transported substrate can be only defined with an accurate biochemical characterization of the proteins, it is possible to speculate that diNPFs could have evolved the capability to transport substrates different than ${\mathrm{NO}}_{3}^{-}$, including hormones and peptides.

Sub-cellular localization of the proteins may be one of the main drivers of both evolution and functional diversity in diNPFs. Although caution is required as the predicted sub-cellular localization of the diNPFs is not experimentally validated, most of the sequences analysed are predicted to the plasma membrane (PM), as is the case of most plant NPFs, which play roles in substrate loading and unloading of cells in different tissues [4] and bacterial POTs, which are mainly involved in the uptake of peptides from the outside [6].

Diatoms have the toolkit to metabolize amino acids and dissolved organic forms of N (DON) and [83] report evidence, even if occasional, of DON uptake by diatoms. Considering that DON concentration in the upper ocean layer is of several micromoles per litre, of which 15–20% are urea or amino acids [83], from one to two orders of magnitude larger than inorganic forms of N in oligotrophic environments, we hypothesize that the transporters predicted to be localized to the plasma membrane could transport alternative sources of N in light- and nutrient-limiting conditions, or hormones, that could mediate biotic interactions, such as diatoms–bacteria relationships [84] or sexual reproduction [85].

The ${\mathrm{NO}}_{3}^{-}$ binding site in the plant AtNPF6.3 crystal structure is represented by a protonated histidine, His356 which forms an electrostatic interaction with ${\mathrm{NO}}_{3}^{-}$ [12,14]. Functional studies have shown that mutating His356 in AtNPF6.3 results in the loss of function [14]. Interestingly, when Tyr370 of ZmNPF6.4 was mutated to His, the protein switched its preference to ${\mathrm{NO}}_{3}^{-}$ over chloride [86]. When His362 was mutated to Tyr in ZmNPF6.6, the protein could not transport ${\mathrm{NO}}_{3}^{-}$ [86]. These functional studies indicate that histidine is essential for ${\mathrm{NO}}_{3}^{-}$ transport. However, His356 is not conserved among AtNPF6.3 orthologues that harbour either a tyrosine or another hydrophobic residue at the corresponding position. diNPFs have a conserved tyrosine in the corresponding position (figure 7; electronic supplementary material, table S2). Another tyrosine (or phenylalanine) from TMH1 is located in close proximity and may contribute to creating a hydrophobic pocket for the substrate (figure 7; electronic supplementary material, table S2). The fact that all diNPFs lack this residue points to a different substrate than ${\mathrm{NO}}_{3}^{-}$ or a different mode of substrate recognition.

Yet, the low range of ${\mathrm{NO}}_{3}^{-}$ conditions could also be compatible with a dual affinity capacity of the diNPFs, as in the case of AtNPF6.3, OsNPF6.5, MtNPF1.3 and ZmNPF6.6 [21–23,86] or with a preserved capacity to transport ${\mathrm{NO}}_{3}^{-}$ even at low range concentrations, a property that has been reported for some NPFs in plants [20]. In the case of AtNPF6.3, the switch between the two different modes of action in response to substrate availability occurs through phosphorylation at threonine residue 101 [87]. We searched for this conserved residue and found it in some of the sequences of diNPFs (electronic supplementary material, table S2).

Additionally, NPFs potential ability to transport ${\mathrm{NO}}_{3}^{-}$ could allow to establish a cross-talk between the availability of ${\mathrm{NO}}_{3}^{-}$ and other signalling pathways, in analogy with plants where some of these transporters play a crucial role also in signalling networks in addition to their transport function, acting as nutrient sensors [88].

Interestingly, a number of sequences were predicted to be located at the vacuole membrane, and all these diNPF sequences belong to the Clade II diNPFs. We therefore hypothesize that Clade II diNPFs are not involved in uptake from the external environment but in re-allocation of ${\mathrm{NO}}_{3}^{-}$ between different cell compartments in response to changing N conditions. This could provide another explanation of the conservation of transporters with low ${\mathrm{NO}}_{3}^{-}$ affinity in diatoms, as inside the cells the concentrations of ${\mathrm{NO}}_{3}^{-}$ can be much higher than in the external environment, reaching in some diatoms even up to 60 mM [78]. This would also be coherent with the lack of correlation between diNPFs gene expression and nutrient availability in the *TARA* Oceans database. In fact, diatoms can dominate phytoplankton communities and outcompete other eukaryotes under fluctuating nutrient conditions, thanks to the presence of large vacuoles for nutrient storage, like ${\mathrm{NO}}_{3}^{-}$, during N repletion periods, and to make it available again from internal structures when in limited N conditions [89]. This storage capacity also plays an important role in relation to light: ${\mathrm{NO}}_{3}^{-}$ can be assimilated into biomass and growth in light and can be stored in the vacuole of diatoms to be respired under darkness and anoxic conditions to gain energy, reducing intracellular ${\mathrm{NO}}_{3}^{-}$ to ${\mathrm{NH}}_{4}^{+}$ through dissimilatory nitrate reduction to ammonium (DNRA) [78,79].

In the future, genetics and biochemistry approaches should be applied to understand diNPFs functional activities, substrate specificity and affinity. Technologies enabling the generation and characterization of overexpressing strains and knockout mutants are in place [90]. Deciphering the complexity of the regulatory networks that control N uptakes and metabolism will help understanding the adaptation of diatoms to N availability in fluctuating intra- and extracellular environments, and it will provide new insights into the ecological success of these microalgae.

## Data accessibility

The data that supports the findings of this study are available in the electronic supplementary material of this article.
